# EEG Channel ‐ Specific Power Variations to differentiate Alzheimer's and Frontotemporal Dementia

**DOI:** 10.1002/alz70860_104123

**Published:** 2025-12-23

**Authors:** Poluri Manaswini, Sajjad Shaik, Debanjali Bhattacharya, Neelam Sinha

**Affiliations:** ^1^ Amrita Vishwa Vidyapeetham, Bengaluru, Karnataka, India; ^2^ Indian Institute of Science (IISc), Bengaluru, Karnataka, India

## Abstract

**Background:**

Electroencephalography (EEG) signal analysis is critical for understanding cognitive decline in neurodegenerative disorders like Alzheimer's Disease (AD) and Frontotemporal Dementia (FTD). The objective of the present study is to identify neural biomarkers for Alzheimer's Disease (AD) and Frontotemporal Dementia (FTD) by independently analyzing EEG channels, brainwave dynamics, and power variations, thereby providing insights into disease‐specific alterations in brain activity and affected brain regions.

**Method:**

Power variations across 19 EEG channels were analyzed using publicly available OpenNeuro EEG dataset to identify altered neural activity regions. Independent sample t‐tests assessed significant differences in band power among AD (*n* = 36), FTD (*n* = 23), and HC (*n* = 29) subjects.

**Result:**

Significant differences in brainwave patterns were observed across EEG channels, corresponding to specific brain regions. Statistically significant channel‐specific power alterations were identified in alpha, beta, and theta bands across groups. Alpha waves, linked to cognitive processing, showed reduced power in posterior channels (Pz, POz, O1) in AD and FTD patients (*p* <0.05), suggesting degeneration in higher‐order cognitive regions. Significant difference in Beta waves were found in frontal (Fz, Fp1, Fp2) and central (Cz, C3, C4) channels with *p* <0.05. Theta waves which are typically elevated in cognitive impairment, reflected disease‐related significant changes in Fz and Cz regions (*p* <0.05). While alpha, beta, and theta bands show significant power alterations across EEG channels due to their direct involvement in cognitive processing, delta and gamma power did not exhibit widespread significant differences due to their functional roles and disease‐specific progression patterns.

**Conclusion:**

EEG analysis reveals localized neural activity changes in specific brain regions in AD and FTD. Brainwave dynamics and channel‐specific power variations serve as biomarkers to distinguish diseased states from healthy functioning.

